# Projections of cancer incidence and cancer‐related deaths in Germany by 2020 and 2030

**DOI:** 10.1002/cam4.767

**Published:** 2016-06-29

**Authors:** Anne S. Quante, Chang Ming, Miriam Rottmann, Jutta Engel, Stefan Boeck, Volker Heinemann, Christoph Benedikt Westphalen, Konstantin Strauch

**Affiliations:** ^1^ Institute of Medical InformaticsBiometry and EpidemiologyChair of Genetic EpidemiologyLudwig Maximilian UniversityMunichGermany; ^2^ Institute of Genetic EpidemiologyHelmholtz Zentrum München – German Research Center for Environmental HealthNeuherbergGermany; ^3^ Munich Cancer Registry (MCR)Munich Tumour Centre (TZM)Institute for Medical Information ProcessingBiometry and EpidemiologyLudwig Maximilian UniversityMunichGermany; ^4^ Department of Internal Medicine IIIHematology and OncologyCampus GroßhadernLudwig Maximilian UniversityMunichGermany

**Keywords:** Breast cancer, cancer deaths projection, cancer incidence projection, colon cancer, prostate cancer

## Abstract

Past patterns of cancer disease and future changes in the demographic structure have a major influence on the projected incidences of human malignancies. In Germany, nearly a quarter of men and 20% of women die of cancer, and it is estimated that in Germany around 51% men and 43% women will develop cancer during lifetime. Here, we project the cancer incidence case number as well as the number of deaths for the most common cancers in the German population for the years 2020 and 2030. By 2030, prostate cancer will be the most common malignancy, surpassing breast cancer. Lung cancer will rank third most frequent cancer and will remain the most common cause of cancer‐related mortality. Additionally, our projections show a marked increase in liver cancer cases with a continuous rise in liver cancer‐related deaths. Finally, we project a constant increase in the incidence of pancreatic cancer. Based on our projections, pancreatic cancer will surpass colorectal and breast cancer to rank as the second most common cause of cancer‐related deaths in Germany by 2030.

## Introduction

As of 2012, breast cancer (70,170), prostate cancer (63,710), colorectal cancer (62,230) and lung cancer (52,520) rank highest in terms of incidence case numbers of malignancies in Germany 1. Accordingly, these entities have been in the focus of funding agencies, the pharmaceutical industries, and preventive programs. Germany has witnessed a major demographic shift over the last decades. Changes in the population structure influence the projections of cancer cases in the future. For the United States, Smith and coworkers projected a significant increase in cancer incidences for the years 2020 and 2030. This projection was based on the fact that there are sociodemographic changes ongoing in the United States, such as a dramatic increase in the number of elderly citizens and minorities 2. Recently, Rahib and colleagues took a similar approach and expanded the projections made by Smith et al. Rahib et al. included not only demographic changes, but also changes in age‐standardized incidence rates by cancer type 3. Based on these variables, different projections of future cancer incidence and deaths were made. Rahib et al. projected an unexpected and alarming rise of thyroid, liver, and pancreatic cancer, the latter becoming the second most frequent cause of cancer‐related deaths around 2021. International publications of similar projections for Germany are rare 4, 5, 6, 7, 8; therefore, such projections could add substantial weight to future global health initiatives. Accordingly, we set out to adapt and improve the projection model established by Rahib and colleagues to calculate the future cancer burden in Germany.

## Materials and Methods

### Data preparation

For this study, cancer registry data from the Robert Koch Institute 9 and demographic projections from the Federal Statistical Office of Germany (published 28 April 2015) were used 10. Within the Robert Koch Institute, the German Centre for Cancer Registry Data are responsible for pooling and quality assurance of the data from population‐based cancer registries in each of the German federal states. These data were retrieved by database query (http://www.krebsdaten.de).

Age‐standardized incidence rates and age‐specific incidence rates, as well as the case numbers for the 15 most common cancer sites (ICD 10 classification) were provided for the years 1999–2011. Age‐standardized mortality rates and age‐specific mortality rates, as well as the death numbers for the 15 most common malignancies were provided for the years 1998–2012. Rates were standardized using the old European Standard Population. Age‐specific rates for each site were grouped by sex and 5‐year age group (from 0 to 84 or 85+ years). Both age‐standardized and age‐specific rates were computed per 100,000 individuals. The standard errors of the age‐standardized rates, which are required for the trend analysis (joinpoint regression) described below, were calculated from the corresponding age‐specific rates and age‐specific population sizes by means of a SAS program (SAS version 9.4, Cary, NC).

Population projections for 2020 and 2030 were provided by the Federal Statistical Office of Germany, which is responsible for collection, processing, and analysis of statistical information concerning economy, society, and environment. Eight different population projections were provided. Here, the G1‐L1‐W1 projection was used, assuming a birth rate of 1.4 children per woman, life expectancy at birth in 2060 of 84.8 years for men and 88.8 years for women, as well as a long‐term net migration of 100,000 individuals per year.

### Trend analysis

To analyze changes in cancer incidence and mortality, age‐standardized cancer incidence rates from 1999 to 2011 and age‐standardized cancer mortality rates from 1998 to 2012 were modeled into joinpoint regression, which is applied to detect points of changing trends (joinpoints) and determine the linear trends between these points. For the calculations, we used a freely available software tool from the National Cancer Institute 11, 12. The joinpoint software selected the best‐fitting trend pattern, that is, the number of time points within the range of years at which the linear trend changes, for each cancer site. The average annual percent change (AAPC) was calculated by summarizing the trend over the time period as weighted average of the annual percent changes (APCs). The AAPC calculations were done for the entire age range, that is, not specific for age groups. This approach was taken because the numbers of cancer‐site‐specific incident cases (or deaths) are rather small (less than 100 or even less than 10 cases) for many age‐specific groups of a 5‐year age range. Therefore, performing the estimation for such rather small age‐specific groups would lead to a considerable variance and instability in the age‐group‐specific AAPC(j) estimates, which is likely to outweigh the apparent advantage of an age‐specific AAPC modeling.

### Projected cancer incidence

Cancer incidences were projected based on the methods applied by Rahib and colleagues 3. Based on the assumption that the pattern of changes in cancer incidence and death rate remain consistent into the future, these projections combine past AAPCs with present age‐specific numbers of incident cases or deaths and projected demographic developments. In contrast to Rahib et al., AAPCs in incidence rates that were not significantly different from zero were still used in the projections in order to avoid any bias. The following formulas were used to project incidences for 2020 and 2030.

$$\text{For}\;{\text{AAPC}}_{i}>0:\;\#\;\text{of Cases}=I_{d}{\left(\frac{{\text{AAPC}}_{i}}{100}+1\right)}^{n}$$

$$\text{For}\;{\text{AAPC}}_{i}<0:\;\#\;\text{of Cases}=\frac{I_{d}}{{\left(\frac{|AAPC_{i}|}{100}+1\right)}^{n}}$$

*AAPC*_*i*_ was calculated by the joinpoint software 12 for each cancer site by sex. The variable *n* equals the adjustment in years, which is 9 for 2020 and 19 for 2030, respectively. *I*_*d*_ is the incidence based on the projected demographics 10 for 2020 and 2030, which equals the sum of the age‐specific incidence rate of year 2011 for each age group *j* multiplied by the corresponding age‐specific population of 2020 and 2030.

$$I_{d}=\sum_{j=x}^{y}\text{Incidence}\;{\text{rate}}_{2011,j}\;\cdot {\text{Population}}_{2020\;or\;2030,j}$$

### Projected cancer‐related deaths

To project cancer‐related deaths, the same algorithm as for incidence projections was applied, with the difference that the mortality data were collected for the period from 1998 to 2012. This approach differs from the methods applied by Rahib and colleagues 3. More specifically, age‐specific mortality rates of year 2012 were used to calculate *D*_*d*_. The adjustment *n* was changed accordingly to 8 and 18 years for 2020 and 2030, respectively. The following formulas were used to project the number of cancer‐related deaths by 2020 and 2030.

$$\text{For}\;{\text{AAPC}}_{d}>0:\;\#\;\text{of Deaths}=D_{d}\cdot {\left(\frac{{\text{AAPC}}_{d}}{100}+1\right)}^{n}$$

$$\text{For}\;{\text{AAPC}}_{d}<0:\;\#\;\text{of Deaths}=\frac{D_{d}}{{\left(\frac{|AAPC_{d}|}{100}+1\right)}^{n}}$$

$$D_{d}=\sum_{j=x}^{y}\text{Mortality rate}{\;}_{2012,j}\;\cdot {\text{Population}}_{2020\;or\;2030,j}$$

## Results and Discussion

### Cancer incidence

**Figure 1:**
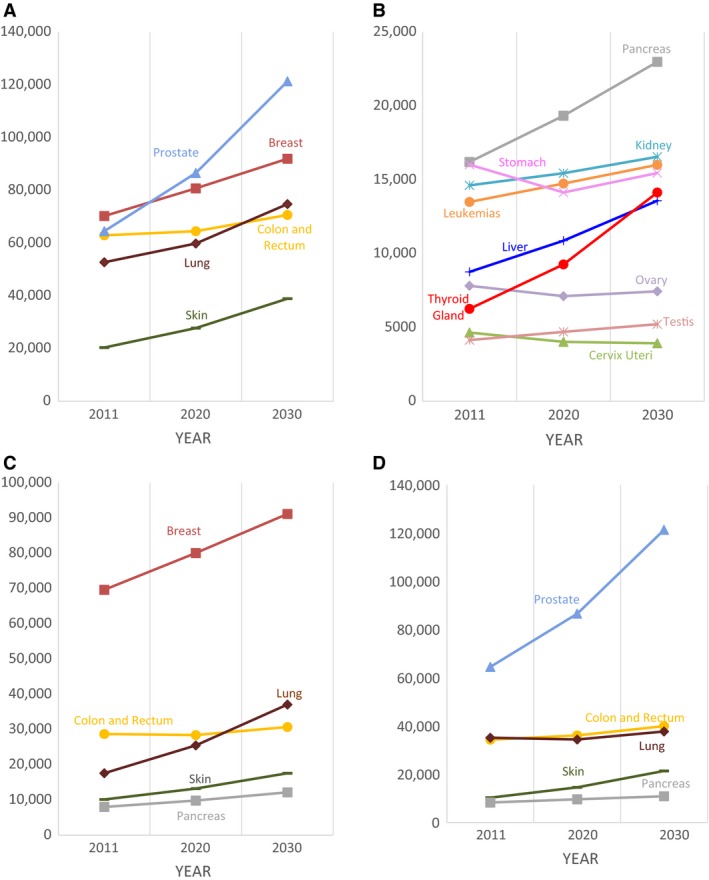
Projected incident cancer cases in Germany by 2020 and 2030. (A) Projected case numbers for the five most common cancers in both sexes. (B) Projected case numbers for the 6th to 14th most common cancers in both sexes.(C) Top five projected case numbers in females.(D) Top five projected case numbers in males.

Table 1 and Figure 1 depict the projected incidences in 2020 and 2030, based on changing demographics and the change in AAPCs for the 14 most common cancer sites in women, men, and combined for both sexes. In both sexes combined (Fig. 1 A and B), the incidence of most cancers is projected to increase until 2030. Based on our projections, prostate cancer will surpass breast cancer, becoming the most frequent malignancy by 2030. Furthermore, lung cancer will surpass colorectal cancer, thus ranking third most common cancer by 2030. Thyroid cancer will increase notably, exceeding liver and ovary cancer to become the 10th most frequent entity in 2030.

**Table 1:** Incidence projections based on demographic changes and AAPCs in incidence rates

Cancer sites	Men		Women		All
	AAPC	Number of cases	AAPC	Number of cases	Number of cases
All cancer sites (C00‐C97 w/o C44)					
2011	0	255,318	0.5	228,259	483,577
2020		283,976		254,360	538,336
2030		312,597		280,876	593,473
Breast (C50)					
2011	0.5	594	1	69,663	70,257
2020		703		80,077	80,780
2030		818		91,200	92,018
Cervix uteri (C53)					
2011			**−1.8**	4647	4647
2020				4023	4023
2030				3924	3924
Colon and rectum (C18‐C21)					
2011	−0.8	34,276	**−1.1**	28,695	62,971
2020		36,104		28,419	64,523
2030		39,938		30,696	70,634
Kidney (C64)					
2011	−0.5	9034	0	5593	14,627
2020		9441		6003	15,444
2030		10,168		6390	16,558
Leukemia (C91‐C95)					
2011	0.2	7533	0.2	5965	13,498
2020		8477		6266	14,743
2030		9525		6494	16,019
Liver (C22)					
2011	1.1	5764	**1.6**	2994	8758
2020		7119		3758	10,877
2030		8781		4803	13,584
Lung (C33‐C34)					
2011	−1.5	35,141	**3.3**	17,576	52,717
2020		34,374		25,451	59,825
2030		37,745		37,056	74,801
Malignant melanoma of skin (C43)					
2011	3.2	10,247	2.8	10,101	20,348
2020		14,541		13,179	27,720
2030		21,358		17,569	38,927
Ovary (C56)					
2011			**−1.7**	7819	7819
2020				7116	7116
2030				7447	7447
Pancreas (C25)					
2011	0.3	8240	**1.3**	7970	16,210
2020		9552		9789	19,341
2030		10,831		12,156	22,987
Prostate (C61)					
2011	**2.1**	64,515			64,515
2020		86,551			86,551
2030		121,335			121,335
Stomach (C16)					
2011	**−2.6**	9573	**−2.7**	6453	16,026
2020		8603		5535	14,138
2030		9485		5974	15,459
Testis (C62)					
2011	**1.9**	4139			4139
2020		4695			4695
2030		5216			5216
Thyroid gland (C73)					
2011	**4.6**	1791	**4.4**	4467	6258
2020		2735		6534	9269
2030		4318		9822	14,140

AAPC, average annual percent change. The bold AAPCs indicated a significant difference from 0 at *α*= 0.05 level.

In women (Fig. 1C), breast cancer is projected to remain the most common malignancy with constant increase in case numbers over the next two decades. Furthermore, lung cancer will surpass colorectal cancer to rank second most frequent malignancy in 2030.

Among men (Fig. 1D), prostate cancer will remain the most common cancer and will continuously increase in incidence over the next two decades. While cases of colorectal and lung cancer will remain fairly stable, pancreatic cancer will surpass kidney and stomach cancer to become the fifth most common cancer by 2030 (Table 1).

### Cancer‐related deaths

**Figure 2:**
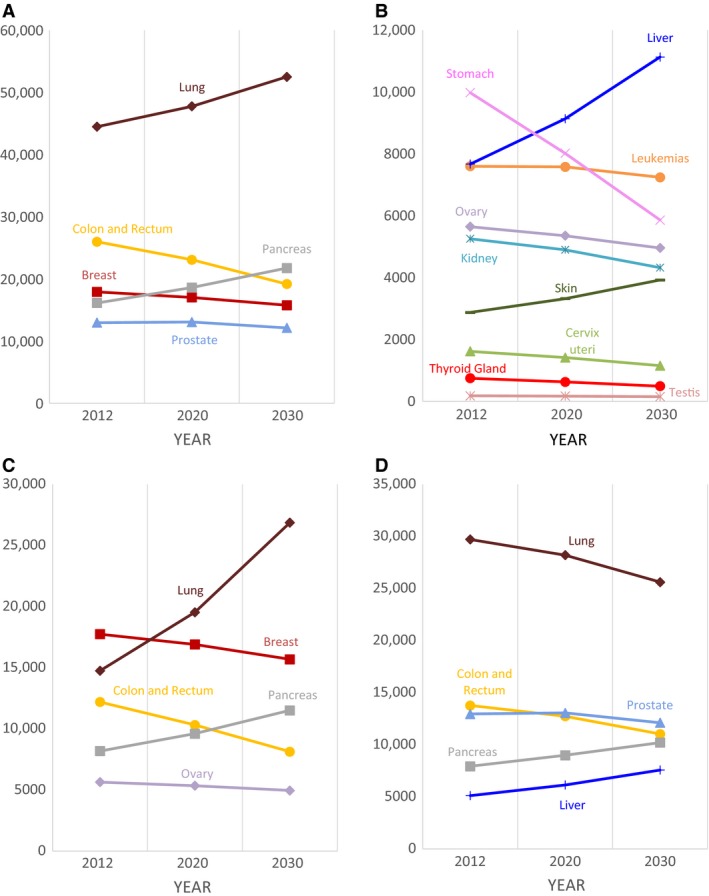
Projected cancer death numbers in Germany by 2020 and 2030. (A) Projected cancer death numbers for the five most common cancers in both sexes. (B) Projected cancer death numbers for the 6th to 14th most common cancers in both sexes. (C) Top five projected cancer death numbers in females. (D) Top five projected cancer death numbers in males.

Projections for cancer‐related deaths caused by the 14 most frequent malignancies in women and men for the years 2020 and 2030 can be found in Table 2 and Figure 2. Overall (Fig. 2A and B), lung cancer will remain the major cause of cancer‐related mortality and the number of deaths will steadily increase until 2030. By 2030, pancreatic cancer will surpass colorectal and breast cancer to rank second most common cause of cancer‐related deaths. Our calculations project a sharp increase in deaths due to liver cancer (sixth), surpassing stomach cancer, which will constantly decrease and fall behind leukemia (seventh) to rank eighth most common cause of cancer‐related deaths by 2030.

**Table 2:** Death projections based on demographic changes and AAPCs in death rates

	Men		Women		All
Cancer sites	AAPC	Number of deaths	AAPC	Number of deaths	Number of deaths
All cancer sites (C00‐C97 w/o C44)					
2012	**−1.8**	119,717	**−1.2**	101,206	220,923
2020		117,780		99,835	217,615
2030		110,119		96,119	206,238
Breast (C50)					
2012	**−5.4**	150	**−1.5**	17,748	17,898
2020		111		16,903	17,014
2030		72		15,689	15,761
Cervix uteri (C53)					
2012			**−2.3**	1617	1617
2020				1416	1416
2030				1153	1153
Colon and rectum (C18‐C21)					
2012	**−2.7**	13,772	**−3.4**	12,200	25,972
2020		12,743		10,319	23,062
2030		11,035		8137	19,172
Kidney (C64)					
2012	**−2.3**	3125	**−2.3**	2131	5256
2020		2963		1937	4901
2030		2624		1699	4323
Leukemia (C91‐C95)					
2012	**−1.4**	4155	**−1.6**	3445	7600
2020		4263		3317	7581
2030		4149		3092	7241
Liver (C22)					
2012	**1**	5117	**0.9**	2553	7670
2020		6140		2992	9133
2030		7581		3551	11,132
Lung (C33‐C34)					
2012	**−2**	29,713	**2.6**	14,752	44,465
2020		28,202		19,542	47,745
2030		25,620		26,870	52,490
Malignant melanoma of skin (C43)					
2012	**1.1**	1627	0.3	1248	2875
2020		1956		1366	3321
2030		2421		1503	3925
Ovary (C56)					
2012			**−1.5**	5646	5646
2020				5356	5356
2030				4959	4959
Pancreas (C25)					
2012	**0.2**	7936	**0.9**	8184	16,120
2020		8992		9612	18,603
2030		10,209		11,517	21,726
Prostate (C61)					
2012	**−2.3**	12,957			12,957
2020		13,059			13,059
2030		12,120			12,120
Stomach (C16)					
2012	**−4.1**	5770	**−4.4**	4208	9978
2020		4764		3257	8020
2030		3545		2313	5859
Testis (C62)					
2012	−1.1	179			179
2020		173			173
2030		154			154
Thyroid gland (C73)					
2012	−2.6	330	**−4.4**	419	749
2020		303		326	629
2030		259		232	491

AAPC, average annual percent change. The bold AAPCs indicated a significant difference from 0 at *α*= 0.05 level.

In women (Fig. 2C), lung cancer will surpass breast cancer (second) to become the most frequent cause of female cancer deaths. Pancreatic cancer will exceed colorectal cancer (fourth) to rank third most common cause of cancer‐related deaths in female patients.

In men (Fig. 2D), numbers of cancer‐related deaths are fairly stable with the exception of pancreatic cancer, liver cancer, and melanoma (Table 2). Lung cancer will remain the most frequent cause of cancer‐related deaths in men, albeit with a projected decrease toward 2030. Deaths due to pancreatic cancer are constantly rising, approaching the numbers of colorectal cancer (third).

In this study, we used a combination of demographic projections, present numbers of incident cases or deaths, and weighted annual percent changes to project cancer incidence and deaths in Germany in the future. Such projections are based on several assumptions and thus prone to systematical errors. First, demographic projections are based on current developments and past trends. Hence, in addition to the projection G1‐L1‐W1, as a sensitivity analysis we have also modeled cancer incidences and death rates based on the G2‐L2‐W2 assumption (birth rate 1.6 children per woman, life expectancy at birth in 2060 of 86.7 years for men and 90.4 years for women, long‐term net migration 200,000 individuals per year), which leads to the same conclusions that were obtained using G1‐L1‐W1 (data not shown). Accordingly, we assume that moderate variations in demographic assumptions, such as the recent changes in immigration, are not strong enough to substantially alter the projected disease patterns.

Second, we, as others, assumed that AAPCs in incidence and death rates will remain constant until 2030. While this seems far‐fetched at first sight, we based our AAPC calculations on an expanded time period of 12 years for incidence and 14 years for mortality. Therefore, we are confident that future trends are in line with the development in the past, at least approximately. Nevertheless, while upcoming interventions such as improved cancer prevention and novel screening modalities can have a major influence on future incidence and mortality, they cannot be modeled realistically with current knowledge.

### Projected trends in different entities

There are several interpretations and explanations for the projections presented here. Prostate cancer is a disease of the elderly, and thus one of the underlying reasons for the increase in incidence is the aging German population. However, there are two forms—indolent and aggressive prostate cancer. Population screening behaviors can appreciably affect incidence rates in discovering more indolent than aggressive disease, but have relatively less effect on mortality, which is largely due to aggressive disease. Thus, in spite of a high prevalence, mortality due to prostate cancer will remain fairly stable 13. Breast cancer has been on the rise over the past decades. Based on our projections this trend will continue. Accordingly, breast cancer will remain the most common cancer in women and the second most common cancer averaged over both sexes. Nevertheless, early detection as well as improved management has led to an improvement in the prognosis of patients suffering from breast cancer 1. Tobacco smoking is the major risk factor for lung cancer. Smoking among women has steadily increased since the 1960s. Accordingly, the projected increase in female lung cancer cases and deaths is the reflection of this trend 14. The projected stabilization in incidence and decrease in deaths due to colorectal cancer are reflecting both improved screening modalities and treatment options even for patients with advanced disease 15. Primary cancers of the liver, including hepatocellular carcinoma and intrahepatic cholangiocarcinoma, are a global health burden. Accordingly, the projected increase in these entities is alarming because they are usually detected at an advanced state and curative treatment options are limited 16, 17. Germany has seen a 50% increase in liver cancers over the past 30 years, which might be attributed not only to known risk factors, such as liver cirrhosis due to chronic hepatitis or alcohol consumption, but also to the emergence of nonalcoholic steatohepatitis (NASH) caused by overweight and obesity 18. Finally, in accordance with projections by Rahib and colleagues 3, we expect the same dramatic increase in pancreatic cancer‐related deaths in Germany by 2030. Pancreatic ductal adenocarcinoma (PDAC) remains an almost uniformly lethal disease with a 5‐year survival rate in the single digits. Although two landmark trials within the last 5 years yielded positive results for patients with metastatic PDAC, the overall prognosis remains dismal 19. Aging of the population, as well as modifiable risk factors such as smoking and obesity, only partly account for the growing number in pancreatic cancer cases and the consecutive deaths due to the disease 20. It has been suggested that PDAC has a long latency between initiation and metastatic potential 21. Thus, early detection of pancreatic cancer might help to offer potentially curative surgery for a larger number of patients. In this light, a recent publication on exosomes as an early and specific marker identifying patients with pancreatic cancer is of great interest 22. Nevertheless, novel diagnostic and therapeutic modalities as well as scientific efforts are desperately needed to meet the upcoming challenge projected here.

Recently, Rahib and colleagues reported incidence and death rates based on AAPCs for the United States 3. While we modified and applied their methodical approach, used consistent methods to project deaths similar to incidence case numbers, and also decided to include nonsignificant AAPCs in our projections. In contrast, Rahib and coworkers set these AAPCs to zero. Accordingly, their projections for cancers with nonsignificant AAPCs are just based on demographic changes. Furthermore, and in contrast to the above‐mentioned authors, we have used longer time periods (12 years for incidence and 14 years for mortality) to calculate the AAPCs.

Nevertheless, we project the same alarming increase in pancreatic cancer‐related deaths for Germany as Rahib et al. previously found for the United States. Furthermore, we project a similar growing number of deaths for liver cancer, although this trend does not reach the magnitude of the projection for the U.S. This is most probably due to some differences between the United States and Germany, weaker rises (1% per year for men and 0.9% per year for women) in terms of AAPCs and a comparatively low baseline value (7670 deaths in 2012). Similarly, our projections show a marked increase in the incident case number of thyroid cancers. However, possibly due to the comparatively low baseline value and differences in the population structure, this increase in thyroid cancer incidence is not as pronounced as in the United States.

In the past, a couple of projections based on demographic changes estimating the future cancer burden in Germany have been published 4, 5, 6, 7, 8. However, most of these projections were not based on past trend changes in cancer incidence and mortality 5, 7, 8. Haberland et al. 4 combined past trend changes in cancer mortality with projections on estimated changes in future demographics for the five most common cancers. The investigators predicted lower numbers of cancer‐related deaths for 2020, most probably because different methods and older data versions were used. Although the study by Haberland et al. used log‐linear models to capture the past trend in cancer incidence and mortality, we decided to perform joinpoint regression, which allows for periods having different trends during the past. These stepwise trends are assumed to be linear on a logarithmic scale rather than modeled as quadratic polynomials as in the approach taken by Haberland et al. 4. We believe that a stepwise linear fit of past trends, followed by averaging the annual percent change over the different periods, is likely to lead to a more realistic extrapolation of incidence and mortality rates into the future than employing a single log‐linear fit over the entire period in one single step that includes quadratic terms, because overfitting is more likely to occur. In concordance with our findings, Haberland et al. also showed the increase in lung cancer deaths in women and decrease in stomach and colon cancer deaths in women and men. Nowossadeck et al. 6 combined past trend changes in cancer incidence and changes in future demographics for two cancer locations up to 2020 using only slightly older datasets than the data underlying this study. Their projected numbers for colon and lung cancer are similar to our results. Going beyond these two cancer locations, we present the first study in Germany with a more comprehensive approach taking 14 different cancer types into consideration.

In summary, we have applied mathematical modeling to project a scenario for the future cancer burden in Germany. While there is a fundamental difference in terms of demographic structure, we see comparable trends to the United States, especially with regard to a considerable rise in pancreatic cancer incidence and mortality. These projections might be helpful to direct scientific and medical efforts to meet the challenges of cancer prevention and treatment in the future.

## Conflict of Interest

None declared.
